# Ubiquitin-specific Protease 35 Promotes Gastric Cancer Metastasis by Increasing the Stability of Snail1

**DOI:** 10.7150/ijbs.87176

**Published:** 2024-01-12

**Authors:** Cunying Ma, Zhuangfei Tian, Dandan Wang, Wenrong Gao, Lilin Qian, Yichen Zang, Xia Xu, Jihui Jia, Zhifang Liu

**Affiliations:** 1 Department of Biochemistry and Molecular Biology, Key Laboratory for Experimental Teratology of Chinese Ministry of Education, School of Basic Medical Sciences, Cheeloo College of Medicine, Shandong University, Jinan, P. R. China.; 2 Department of Microbiology, Key Laboratory for Experimental Teratology of Chinese Ministry of Education, School of Basic Medical Sciences, Cheeloo College of Medicine, Shandong University, Jinan, P. R. China.

**Keywords:** gastric cancer, deubiquitinase, USP35, Snail1, EMT

## Abstract

Deubiquitinase (DUB) dysregulation is closely associated with multiple diseases, including tumors. In this study, we used data from The Cancer Genome Atlas and Gene Expression Omnibus databases to analyze the expression of 51 ubiquitin-specific proteases (USPs) in gastric cancer (GC) tissues and adjacent non-neoplastic tissues. The Kaplan-Meier Plotter database was used to analyze the association of the differentially expressed USPs with the overall survival of patients with GC. The results showed that five USPs (USP5, USP10, USP13, USP21, and USP35) were highly expressed in GC tissues and were associated with poor prognosis in patients with GC. Because the epithelial-mesenchymal transition enables epithelial cells to acquire mesenchymal features and contributes to poor prognosis, we investigated whether these USPs had regulatory effects on the key epithelial-mesenchymal transition transcription factor Snail1. Our results showed that USP35 exhibited the most significant regulation on Snail1. Overexpression of USP35 increased and its knockdown decreased Snail1 protein levels. Mechanistically, USP35 interacted with Snail1 and removed its polyubiquitinated chain, thereby increasing its stability. Furthermore, USP35 promoted the invasion and migration of GC cells depending on its DUB activity. USP35 knockdown exhibited the opposite effect. Snail1 depletion partially abrogated the biological effects of USP35. Experiments using nude mouse tail vein injections indicated that wild-type USP35, but not the catalytically inactive USP35-C450A mutant, dramatically enhanced cell colonization and tumorigenesis in the lungs of mice. In addition, USP35 positively correlated with Snail1 expression in clinical GC tissues. *Helicobacter pylori* infection increased USP35 and Snail1 expression levels. Altogether, we found that USP35 can deubiquitinate Snail1 and increase its expression, thereby contributing to the malignant progression of GC. Therefore, USP35 may serve as a viable target for GC treatment.

## Introduction

Gastric cancer (GC) is one of the most common malignancies worldwide and the fourth leading cause of cancer-related deaths 1, 2. *Helicobacter pylori* infection contributes to chronic gastritis and gastric ulcers and is an important risk factor for stomach neoplasms 3. Despite the relatively long survival period of early GC, most patients diagnosed at an advanced stage with lymph node metastasis have a poor prognosis and short overall survival (OS) 3-4. Therefore, exploring the mechanisms of invasion and metastasis and identifying novel therapeutic targets for GC are crucial.

Epithelial-mesenchymal transition (EMT) allows epithelial cells to lose their epithelial phenotype and acquire mesenchymal properties that enable invasion and metastatic dissemination 5. EMT is critical for both embryonic development and the metastasis of cancer cells from the primary tumor to distant sites 6. Accumulating evidence suggests that EMT in tumors leads to poor clinical prognosis 5, 7, 8. During EMT, mesenchymal markers, such as N-cadherin and Vimentin, are upregulated, whereas the epithelial markers, usually E-cadherin, are downregulated 9. This process is regulated by a wide range of EMT-inducing transcription factors, especially Snail1/2, Twist1/2, and Zeb1/2 10, 11, 12.

As a core driver of EMT, the Snail protein family member Snail1 is highly unstable and can be quickly degraded via the ubiquitin-mediated proteasome pathway. Multiple ubiquitin E3 ligases, including FBXO11 13, β-TrCP 14, FBXL5 15, Pellino-1 16, FBXL14 17, FBXW7 18, and PPIL2 19, have been shown to facilitate the ubiquitination and degradation of Snail1. Our previous study demonstrated that the F-box protein FBXO31 suppresses EMT and metastasis of GC through ubiquitin-mediated proteasomal degradation of Snail1 20. Ubiquitination can be reversed by various deubiquitinases (DUBs) responsible for removing ubiquitin chains from target substrates and maintaining protein stability. Several DUBs, such as DUB3 21, 22, USP27X 23, OTUB1 24, USP29 25, 26, USP9X 27, USP37 28, and USP13 29, have been reported to deubiquitinate Snail1 and increase its stability. In this study, we validated that USP35 can remove the polyubiquitination chain from Snail1 and increase its expression in GC cells.

As a member of DUB, USP35 participates in several physiological and pathological processes by deubiquitinating its target substrates. Park et al. reported that USP35 deubiquitinates Aurora B and maintains its stability to regulate mitotic progression 30. Wang et al. demonstrated that USP35 stabilizes RRBP1 and alleviates endoplasmic reticulum stress-induced apoptosis in non-small cell lung cancer cells 31. Additionally, USP35 regulates ferroptosis and cisplatin-induced apoptosis by directly interacting with and stabilizing ferroportin 32 and BIRC3 33. In ovarian cancer, USP35 deubiquitinates STING and affects STING-mediated interferon signaling 34. However, the role of USP35 in EMT and tumor metastasis remains unclear.

In the current study, we found that the expression of USP35 was increased in GC tissues and was associated with nodal metastasis and tumor grade. *H. pylori* infection may contribute to the upregulation of USP35 in GC. Higher USP35 expression resulted in a poorer prognosis in patients with GC. USP35 promoted GC cell metastasis by deubiquitinating and stabilizing Snail1. In addition, USP35 was positively associated with Snail1 expression in tumor cell lines and human GC tissues. Our findings substantiate the regulatory effect of USP35 on the malignant progression of GC and defined it as a novel Snail1 deubiquitinase.

## Materials and Methods

### Plasmids and siRNAs

The Myc-tagged USP35 expression vector (Myc-USP35), HA-tagged USP35 expression vector (HA-USP35), HA-tagged USP35 mutant vector USP35-C450A (HA-USP35-C450A), and pLVX-AcGFP1-N1 vectors were generously provided by Professor Zhang Pengju (Shandong University, Jinan, China). The mutated ubiquitin vector, including HA-Ub K0, HA-Ub K6, HA-Ub K11, HA-Ub K27, HA-Ub K29, HA-Ub K33, HA-Ub K48, and HA-Ub K63, were generously provided by Professor Zhao Wei (Shandong University, Jinan, China). The Flag-tagged USP5 expression vector (Flag-USP5) was generously provided by Professor Gao Chengjiang (Shandong University, Jinan, China). Flag-tagged USP13 expression vector (Flag-USP13) (plasmid #61741), Flag-tagged Snail1 expression vector (Flag-Snail1) (plasmid #16218), and HA-tagged Ubiquitin (HA-Ub) (plasmid #18712) were purchased from Addgene (Cambridge, MA, USA). The HA-tagged Snail1 expression vector (HA-Snail1) and Flag-tagged USP35 (Flag-USP35) were constructed by inserting the ORF of Snail1 or USP35 into the HA-tagged or Flag-tagged expression vector. The Myc-tagged USP35-C450A mutant vector and HA-tagged USP35 truncation mutants were constructed by performing point or truncated mutations in the wild-type USP35 expression vector. pLVX-WT-USP35 and pLVX-USP35-C450A were constructed by cleaving the ORF sequences of WT-USP35 or USP35-C450A from Myc-tagged WT-USP35 or USP35-C450A expression vector and inserting these sequences into the pLVX-AcGFP1-N1 vector between the *Eco*R I and *Bam*H I sites. Flag-tagged USP10 expression vector (Flag-USP10) (P44018), Flag-tagged USP21 expression vector (Flag-USP21) (P30769), and SgSnail1 (P28637) were purchased from MiaoLing Plasmid (Wuhan, China). All the constructs were verified by sequencing. The primer sequences used in this study are listed in Supplementary Table S1. siRNA sequences targeting USP35 or the negative control were synthesized by GenePharma (Shanghai, China). The siRNA sequences are listed in Supplementary Table S2.

### Cycloheximide chase assay

MKN-45 GC cells transfected with siRNAs or expression vector were treated with 100 μg/mL cycloheximide (CHX; 239764, Sigma-Aldrich, USA) for the indicated durations. Cellular proteins were extracted and detected using Western blot.

### MG132 treatment assay

MKN-45 GC cells were transfected with USP35 siRNAs or negative control siRNAs for 72 h. Thereafter, the transfected cells were treated with 10 μM MG132 (HY-13259; MedChemExpress, USA) for 6 h. Cellular proteins were extracted and identified using Western blot.

### Lentivirus infection

pLVX-USP35, pLVX-USP35-C450A, or the control vector was transiently transfected into HEK293T cells with psPAX2 and pMD2.G to produce lentiviruses. BGC-823 and MKN-45 cells were infected with different lentiviruses in the presence of HitransG P (REVG005; Genechem, China) and selected in a medium containing 4 μg/mL puromycin (P8230; Solarbio, China).

### Western blot and co-immunoprecipitation assays

Total proteins were extracted from GC cells using RIPA lysis buffer (P0013B; Beyotime, China) containing proteinase inhibitors (HY-K0010; MedChemExpress, USA). Protein concentration was determined using a BCA reagent (P0011; Beyotime, China). After boiling, the proteins were separated by sodium dodecyl sulfate-polyacrylamide gel electrophoresis (SDS-PAGE), transferred onto polyvinylidene difluoride (PVDF) membranes (IPVH00010; Merck Millipore, Germany), and immunoblotted as described previously 35 using the designated antibodies. For the endogenous immunoprecipitation (IP) assay, HEK293T or GC cells were inoculated onto 10-cm culture plates. For the exogenous co-immunoprecipitation (Co-IP) assay, HEK293T and GC cells were seeded onto 10-cm culture plates and transfected with different expression vectors for 48 h. The cells were then lysed using IP lysis buffer (P0013; Beyotime, China) containing proteinase inhibitors. The lysate was incubated with Protein A/G Magnetic Beads (HY-K0202; MedChemExpress, USA) pre-bound with a specific antibody for 30 min at room temperature. The magnetic beads were washed four times with 400 µL of phosphate buffered saline containing Tween-20 (PBST) and re-suspended in 40 µL of 2 × loading buffer. The immunoprecipitated proteins were detected using Western blot. All antibodies used in this study are listed in Table S3.

### Statistical analysis

Data were analyzed by GraphPad Prism v 8.0 2 software using the Student's *t*-test (two-tailed) or two-way ANOVA. Data are expressed as mean ± standard deviation (SD) from at least three independent assays. Pearson's correlation test was used to detect correlations between USP35 and Snail1 expression. Statistical significance was set at p < 0.05.

The other methods and materials are in **Supplementary information.**

## Results

### Identification USP35 as a regulator for Snail1 in GC

**Figure 1:**
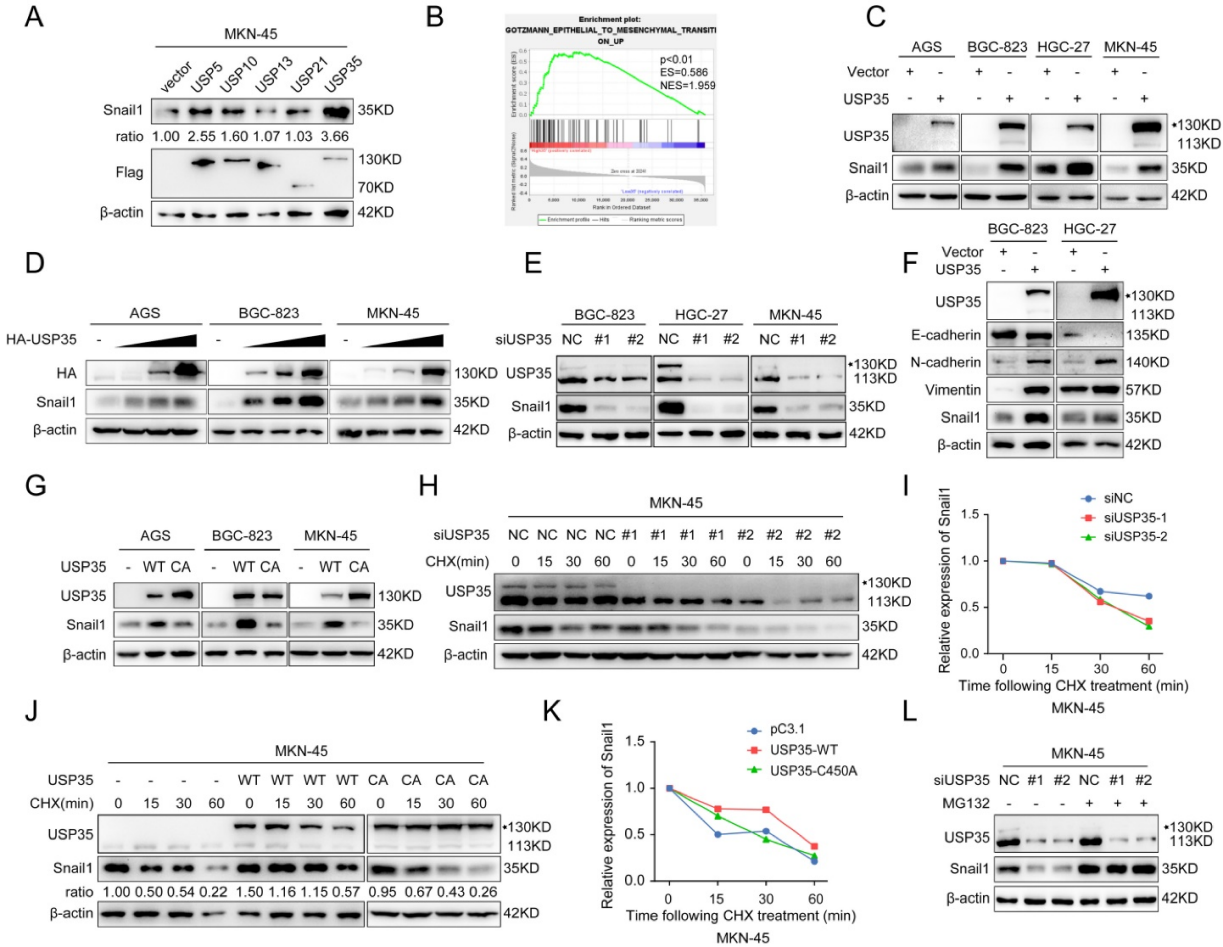
** USP35 increases Snail1 protein levels by maintaining Snail1 stability**. **A.** Western blot detection of Snail1 and Flag expression in MKN-45 cells transfected with Flag-tagged USP5, USP10, USP13, USP21, or USP35. Quantitative analysis of Snail1 protein abundance with ImageJ software. β-actin was used as loading control. B. Gene set enrichment analysis (GSEA) results of the correlation of USP35 mRNA expression with the EMT pathway based on the data of a published cohort (http://www.cbioportal.org/). Red and blue colors indicate high and low levels of USP35, respectively. The barcode plot represents the indexed position of the genes in each gene set. NES, normalized enrichment score; ES, enrichment score. **C-E.** Western blot detection of the indicated gene expression in several GC cells transfected with empty vector or HA-tagged USP35 expression vector (C), an increased concentration gradient of the HA-tagged USP35 expression vector (HA-USP35) (D), negative control siRNA (NC) or two USP35 siRNA (siUSP35) (E). **F.** Western blot detection of E-cadherin, N-cadherin, Vimentin and Snail1 protein levels in GC cells transfected with an empty vector or HA-tagged USP35 expression vector (USP35). **G.** Western blot analysis of Snail1 expression level in different GC cells transfected with the empty vector, WT-USP35 vector (WT), or the USP35-C450A mutant (CA). **H, J.** Western blot detection of endogenous Snail1 degradation ratio in MKN-45 cells transfected with negative control or USP35 siRNA (H) and empty vector, WT-USP35 (WT), or USP35-C450A mutant (CA) (J), and then treated with CHX for the indicated duration. **I, K.** Snail1 protein abundance in H (I) and J (K) as quantified using ImageJ software, normalized to β-actin levels, and statistically analyzed. **L.** Western blot analysis of Snail1 protein in MKN-45 cells transfected with the NC or USP35 siRNA for 72h before treatment with MG132 (10 µM) for 6 h.

We initially investigated the expression of 51 USPs in GC and noncancerous tissues using selected datasets from The Cancer Genome Atlas (TCGA) and Gene Expression Omnibus (GEO) databases and found that 16 USPs were highly expressed in GC tissues in both databases (Supplementary Figs. S1 A, B). We used the Kaplan-Meier Plotter database to investigate the correlation between the 16 USPs and the prognosis of patients with GC. The results indicated that the high expression of five USPs (USP5, USP10, USP13, USP21, and USP35) was closely related to the poor prognosis of patients with GC (Supplementary Fig. S2). Given that invasion and metastasis are the main factors leading to cancer-associated death and that the EMT endows epithelial cells with mesenchymal features to drive the metastatic cascade 5, we determined whether the five USPs have regulatory roles in the expression of the key EMT transcription factor Snail1. As shown in Fig. 1A, USP35 overexpression led to the most significant increase in Snail1 protein levels in MKN-45 cells. Gene set enrichment analysis verified the significant association of USP35 expression with the EMT pathway (Fig. 1B). We investigated whether USP35 regulates other EMT transcription factors, including Slug, Zeb1, and Twist1, and found that USP35 overexpression had no significant effect on their expression levels (Supplementary Fig. S3). To confirm the regulatory effect of USP35 on Snail1, we transfected USP35 expression vectors into several GC cell lines, including AGS, BGC-823, HGC-27, and MKN-45, and estimated the expression level of Snail1. Our results showed that overexpression of USP35 increased the protein level of Snail1 in these GC cells (Fig. 1C) in a dose-dependent manner (Fig. 1D), but USP35 did not change the mRNA level of *Snail1* (Supplementary Fig. S4). In contrast, USP35 knockdown using specific siRNAs reduced the Snail1 protein levels (Fig. 1E). Furthermore, we investigated the regulation of USP35 on EMT markers and found that USP35 overexpression decreased the protein levels of the epithelial marker E-cadherin and increased those of the mesenchymal markers N-cadherin and Vimentin in GC cells (Fig. 1F). Subsequently, we examined the regulatory effect of the enzymatically inactive mutant USP35-C450A on Snail1 protein expression to determine whether the enzymatic activity of USP35 is required for the regulation of Snail1. The results revealed that wild-type USP35 (WT-USP35) dramatically increased Snail1 protein levels, whereas USP35-C450A did not affect Snail1 protein levels, indicating that the enzymatic activity of USP35 is necessary for its regulation (Fig. 1G).

### USP35 maintains Snail1 protein stability in GC

Because USP35 did not affect *Snail1* mRNA expression, we determined whether USP35 regulates the protein stability of Snail1. We added the protein translation inhibitor CHX to the transfected GC cells for different durations and then determined the turnover rate of Snail1. Our results showed that USP35 knockdown by siRNA favored Snail1 degradation in GC cells (Figs. 1H, I), whereas overexpression of WT-USP35 prolonged the half-life of Snail1 and restrained its degradation (Figs. 1J, K). However, the USP35-C450A mutant did not show similar results. We then used the proteasome inhibitor MG132 to treat GC cells transfected with USP35 siRNA. As shown in Fig. 1L, MG132 treatment abrogated USP35 siRNA-induced Snail1 degradation, indicating that USP35 increased Snail1 expression by inhibiting proteasomal degradation.

### USP35 interacts with Snail1

**Figure 2:**
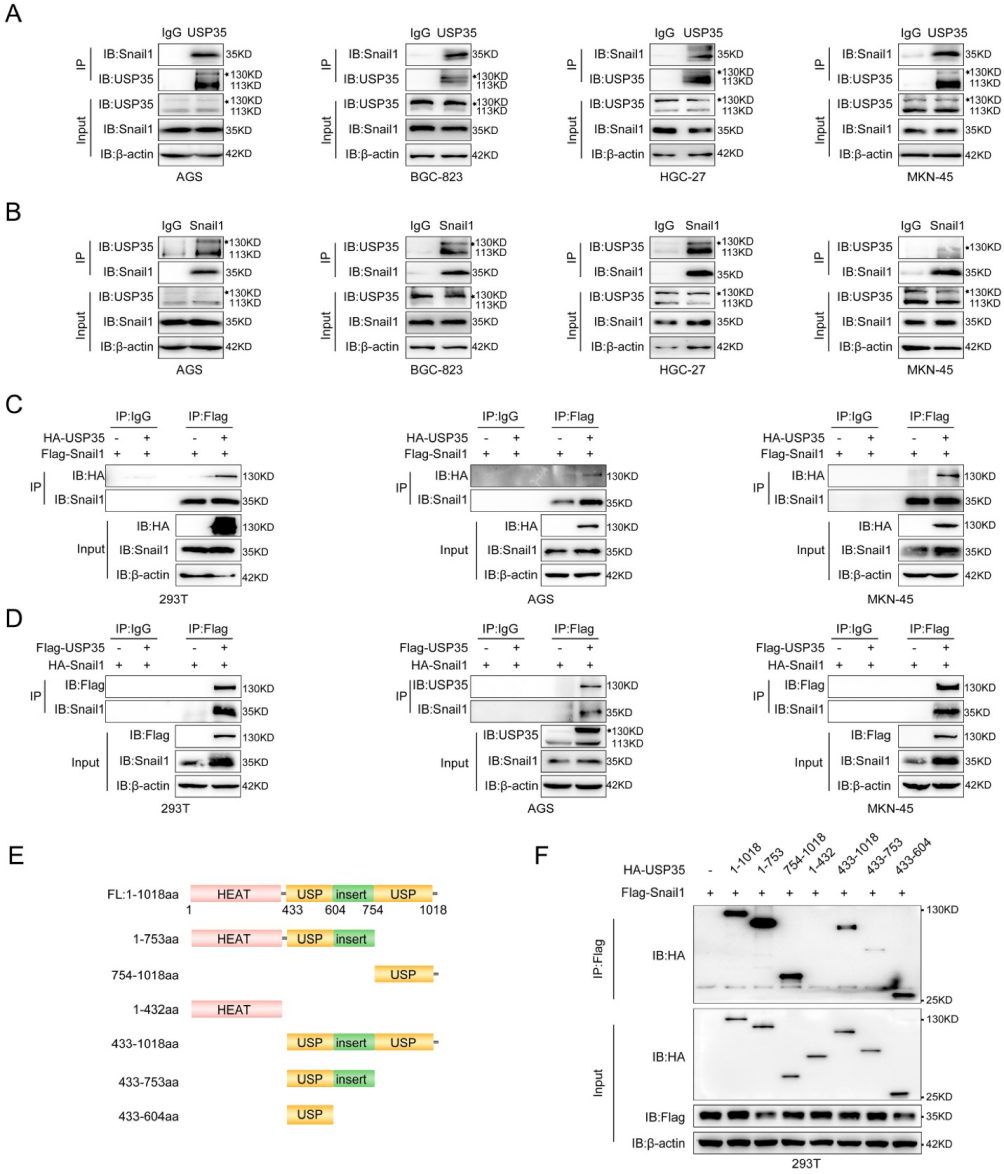
**USP35 interacts with Snail1. A and B.** Detection of endogenous interactions between USP35 and Snail1 in several GC cells by immunoprecipitation (IP) using an anti-USP35 antibody to pull down Snail1 (A) or an anti-Snail1 antibody to pull down USP35 (B). IgG was used as the negative control. **C.** Detection of exogenous interactions between USP35 and Snail1 using a Co-IP assay by adding an anti-Flag antibody to pull down HA-USP35 in HEK-293T, AGS, and MKN-45 cells transfected with Flag-Snail1 in combination with empty vector or HA-USP35. **D.** Detection of exogenous interactions between USP35 and Snail1 using the Co-IP assay by adding an anti-Flag antibody to pull down HA-Snail1 in HEK-293T, AGS, and MKN-45 cells transfected with HA-Snail1 in combination with empty vector or Flag-USP35. **E.** Schematic diagram of USP35 and its truncated mutants. **F.** Detection of the interaction between Snail1 and USP35 truncation mutants using Co-IP assay. The experimental procedure is the same as that described in (C).

We further explored the interaction between USP35 and Snail1 in GC cells using reciprocal IP. As shown in Fig. 2A and 2B, Snail1 could be pulled down by the USP35 antibody, and USP35 could also be pulled down by the Snail1 antibody in several different GC cells. We further transfected Flag-Snail1 and HA-USP35 into HEK293T and GC cells and performed an exogenous Co-IP assay using an anti-Flag antibody.

The results showed that HA-tagged USP35 can co-immunoprecipitate with Flag-tagged Snail1 (Fig. 2C). Similarly, when Flag-USP35 and HA-Snail1 vectors were transfected into HEK293T and GC cells, HA-tagged Snail1 can also co-immunoprecipitate with Flag-tagged USP35 upon addition of a Flag antibody (Fig. 2D). Next, we identified the domain(s) of USP35 that were involved in the interaction with Snail1. According to previous reports, USP35 consists of the N-terminal HEAT repeat region (aa 1-432) and the C-terminal USP catalytic domain (aa 433-1018) 34. Therefore, we constructed a series of truncated mutants (Fig. 2E) and transfected them with Flag-Snail1 into HEK293T cells for the Co-IP assay. As shown in Fig. 2F, deletion of the C-terminal USP domain abrogated the interaction with Snail1, indicating that the USP domain at the C-terminus of USP35 mediated the interaction between USP35 and Snail1.

### USP35 removes the polyubiquitination chain from Snail1

**Figure 3:**
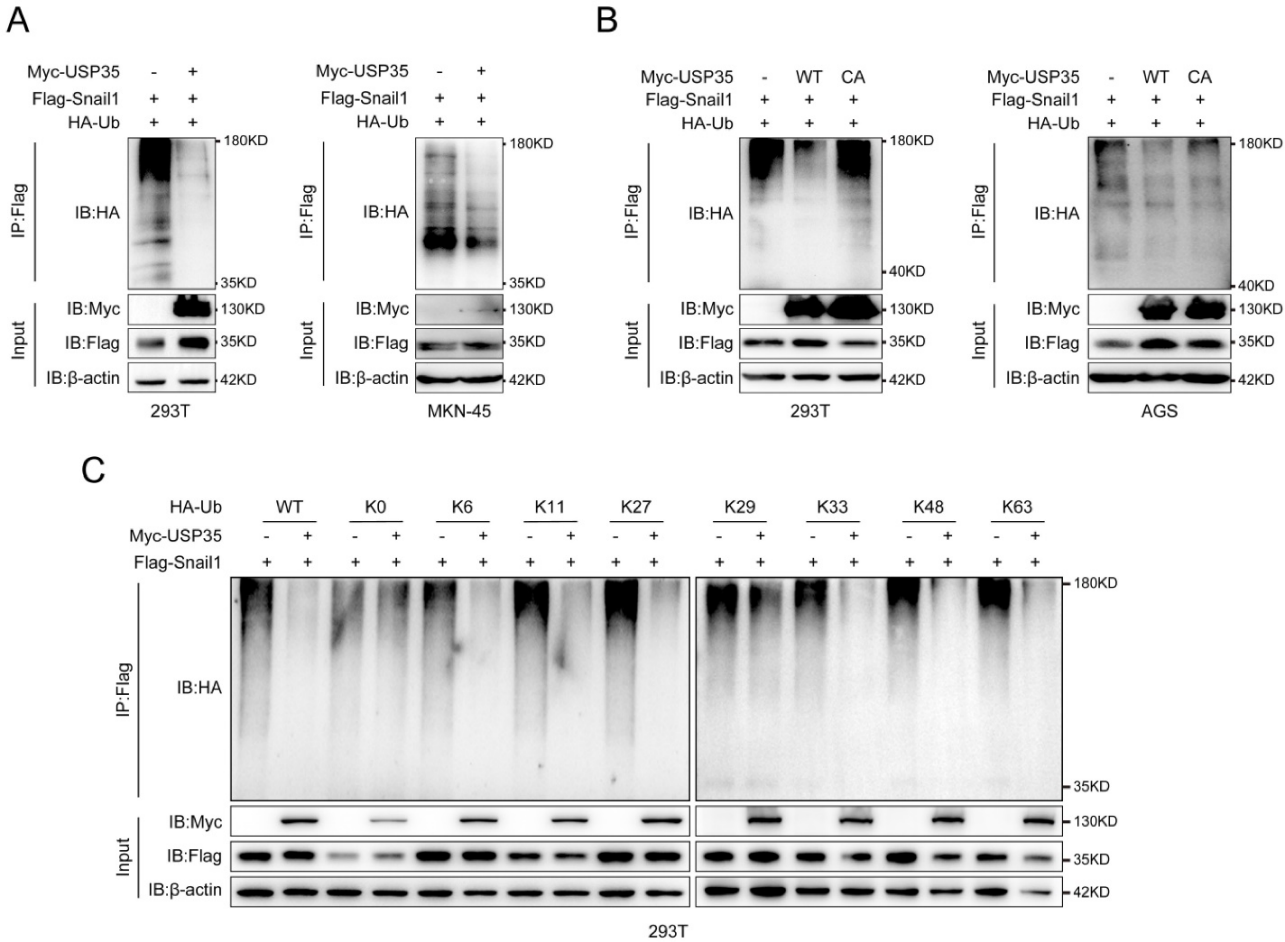
** USP35 deubiquitinates Snail1. A.** IP analysis of the Snail1 ubiquitination in HEK293T and MKN-45 cells transfected with Flag-Snail1 and HA-Ub in combination with the empty vector or Myc-USP35. **B.** IP analysis of the Snail1 ubiquitination in HEK293T and AGS cells transfected with Flag-Snail1 and HA-Ub together with the empty vector, Myc-USP35 (WT), or Myc-USP35 mutant (CA). **C.** Analysis of Snail1 ubiquitination with IP assay in HEK293T cells transfected with Flag-Snail1 and HA-Ub or its lysine residue-modified forms HA-Ub K0, HA-Ub K6, HA-Ub K11, HA-Ub K27, HA-Ub K29, HA-Ub K33, HA-Ub K48, or HA-Ub K63, together with empty vector or Myc-USP35.

Subsequently, we determined whether USP35 deubiquitinates Snail1. We transfected Flag-Snail1 and HA-Ub, together with Myc-USP35 or empty vector, into HEK293T and MKN-45 cells and performed a Co-IP assay. The results shown in Fig. 3A indicate that the ectopic expression of USP35 decreased the polyubiquitination of Snail1. We further transfected Myc-USP35 (WT) or the USP35-C450A mutant (CA) with loss of DUB activity, together with HA-Ub and Flag-Snail1, into AGS and HEK293T cells to determine whether the DUB activity of USP35 is indispensable for the deubiquitination of Snail1. The result showed that the mutant USP35-C450A lost its ability to deubiquitinate Snail1 in AGS and HEK293T cells, indicating that the DUB activity of USP35 is required for the removal of the polyubiquitinated chain from Snail1 (Fig. 3B). It is well known that different forms of ubiquitin chains (K6, K11, K27, K29, K33, K48, and K63) can perform different functions.

Therefore, to investigate the mode of deubiquitination of Snail1 by USP35, we co-transfected Flag-Snail1 with WT HA-Ub, K0 HA-Ub (with all lysine residues in Ub were mutated to arginine), or one among HA-Ub K6, K11, K27, K29, K33, K48, and K63 (in which all lysine residues except those at positions 6, 11, 27, 29, 33, 48, and 63, respectively, were replaced with arginine), together with empty vector or Myc-USP35, into HEK293T cells. Our results indicated that USP35 efficiently removed the K6, K11, K27, K33, K48, and K63-linked polyubiquitination chains of Snail1 (Fig. 3C).

### USP35 facilitates GC progression in a DUB activity-dependent manner

**Figure 4:**
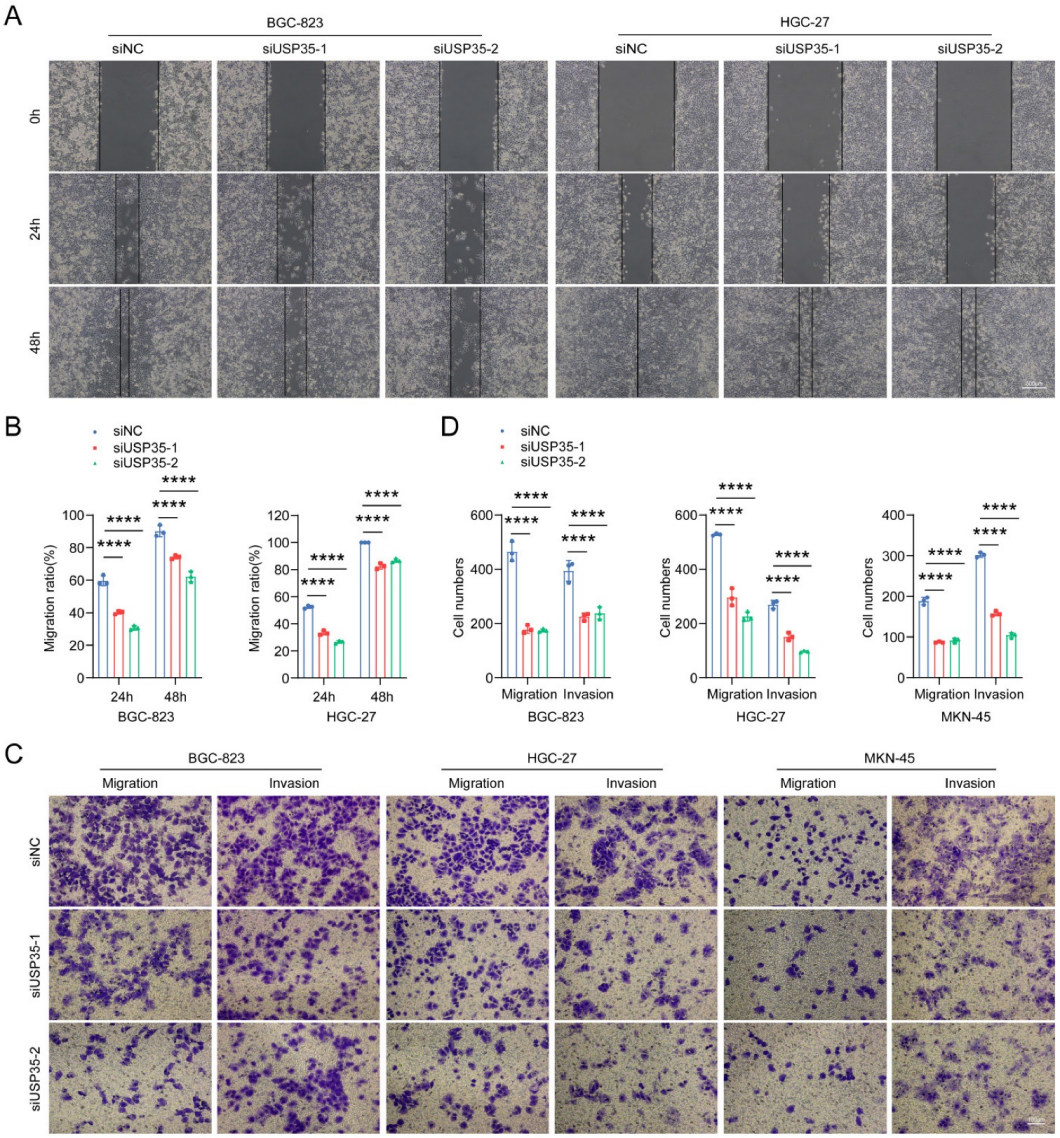
** Depletion of USP35 inhibits GC cell invasion and migration. A.** Representative images of scratch wound healing assay in GC cells transfected with negative control siRNA or USP35 siRNA. Scale bar: 500 µm. **B.** Statistical analysis of the scratch wound healing assay in GC cells transfected with negative control siRNA or USP35 siRNA. **C.** Representative images of the Transwell invasion and migration assays in GC cells transfected with negative control or USP35 siRNA. Scale bar: 100 µm. **D.** Statistical analysis of the cell numbers passing through the Transwell chamber in different transfected GC cells. All data are the mean ± SD, unpaired Student *t-test*. **** *p* < 0.0001.

Then we investigated the biological functions of USP35. Transwell and scratch wound healing assays suggested that USP35 knockdown inhibited the invasion and migration of GC cells (Figs. 4A-D), whereas USP35 overexpression facilitated cell invasion and migration (Supplementary Figs. S5A-D). EdU and CCK-8 assays were used to detect cell proliferation and survival. The results demonstrated that USP35 knockdown with siRNAs remarkably reduced cell proliferation and survival ability (Fig. S6 A-C), whereas overexpression of USP35 promoted cell proliferation and survival (Fig. S6 D-F), which is consistent with the findings that USP35 can promote mitotic progression 30.

**Figure 5:**
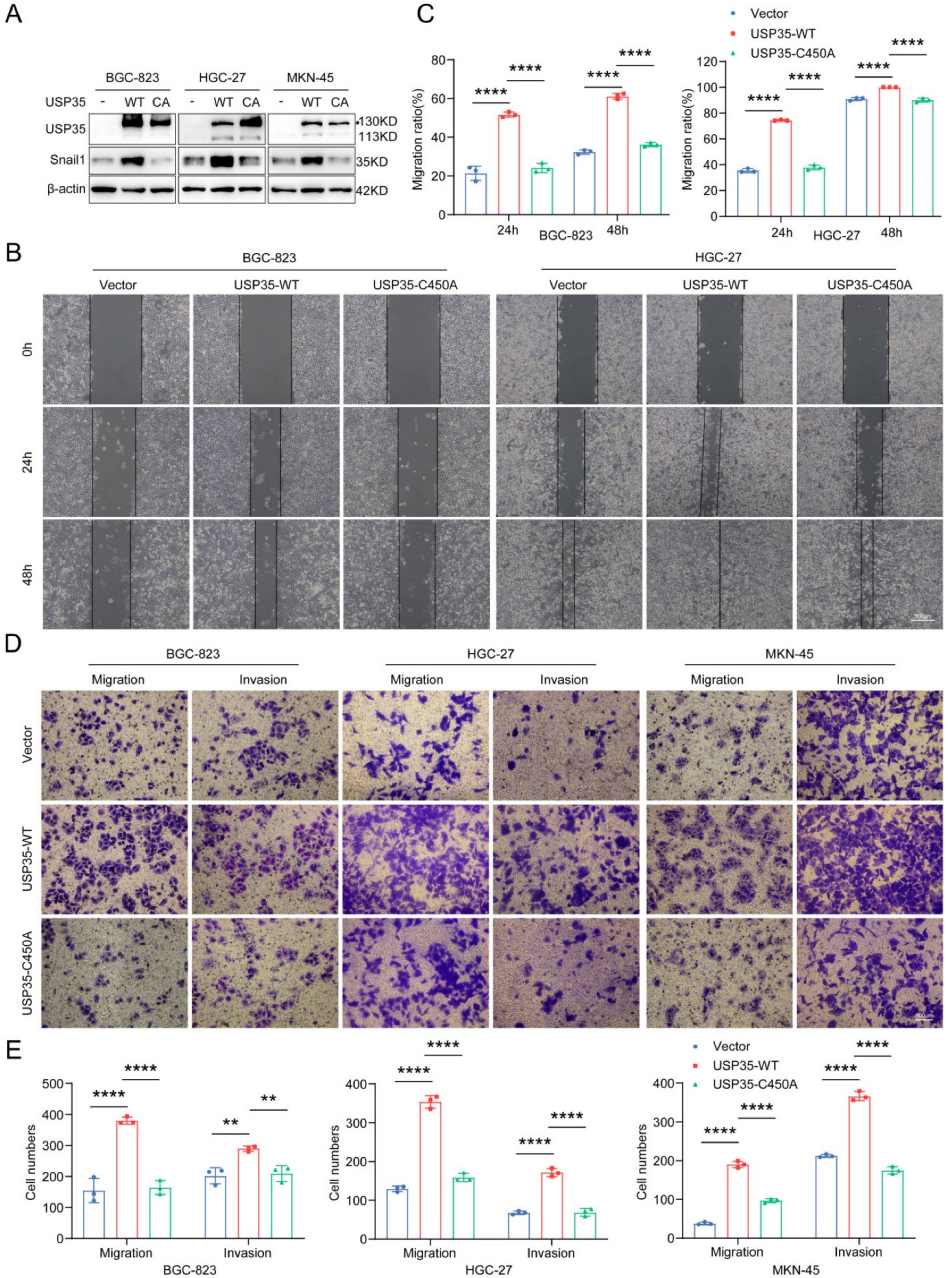
** USP35 overexpression promotes GC cell invasion and migration. A.** Western blot detection of Snail1 and USP35 levels in GC cells transfected with empty vector, WT-USP35 (WT) or USP35-C450A mutant (CA). **B.** Representative images of the scratch wound healing assay in transfected GC cells. Scale bar: 500 µm. **C.** Statistical analysis of cell migration rate in the scratch wound healing assay in transfected GC cells. **D.** Representative images of the Transwell invasion and migration assays in transfected GC cell. Scale bar: 100 µm. **E.** Statistical analysis of the cell numbers passing through the Transwell chamber in transfected GC cells. All data are the mean ± SD, unpaired Student *t-test.* ** *p* < 0.01; **** *p* < 0.0001.

To further investigate whether the enzymatic activity of USP35 is required for its biological effects, we transfected GC cells with a WT-USP35 vector or USP35-C450A mutant (Fig. 5A) and performed Transwell and scratch wound healing assays. As shown in Figs. 5B-E, overexpression of WT-USP35 facilitated cell invasion and migration, whereas overexpression of the mutant USP35-C345A disrupted the biological role mediated by WT-USP35, indicating that the deubiquitinase activity of USP35 is required for its function.

### USP35 regulates GC cell invasion and migration via Snail1

**Figure 6:**
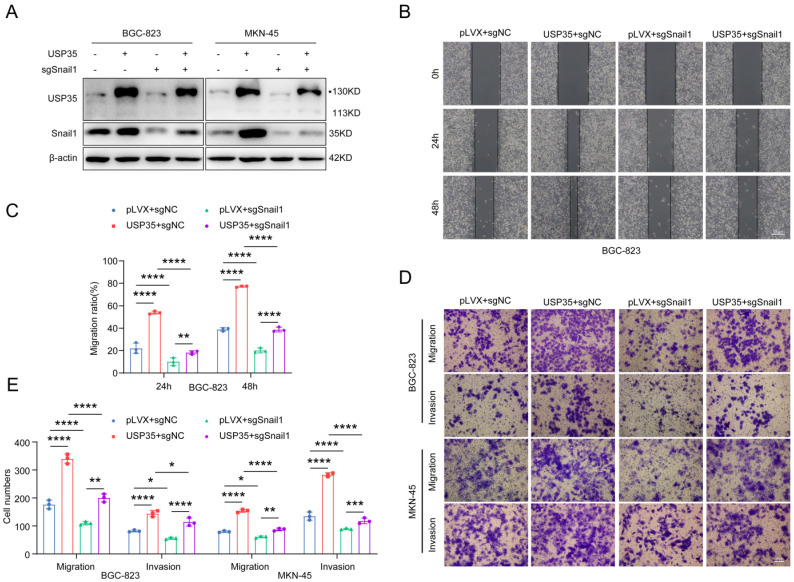
** Snail1 is involved in USP35-mediated GC cell invasion and migration. A.** Western blot detection of Snail1 protein expression in GC cells with different transfections. **B.** Representative images of the scratch wound healing assay of GC cells with different transfections. Scale bar: 500 µm. **C.** Statistical analysis of cell migration rate in the scratch wound healing assay. **D.** Representative images of the Transwell invasion and migration assays of GC cells with different transfections. Scale bar: 100 µm. **E.** Statistical analysis of the cell numbers passing through the Transwell chamber in transfected GC cells. All data are the mean ± SD, unpaired Student *t-test*. * *p* < 0.05; ** *p* < 0.01; *** *p* < 0.001; **** *p* < 0.0001.

We performed rescue experiments to determine whether USP35 promotes GC cell invasion and migration via Snail1. We knocked down Snail1 in GC cells stably transfected with USP35 (Fig. 6A) and assessed their invasion and migration abilities. As shown in Figs. 6 B-E, Snail1 knockdown abolished USP35 overexpression-induced GC cell invasion and migration. These results suggest that USP35 regulates GC cell invasion and migration via Snail1.

### USP35 promotes GC metastasis *in vivo*

**Figure 7:**
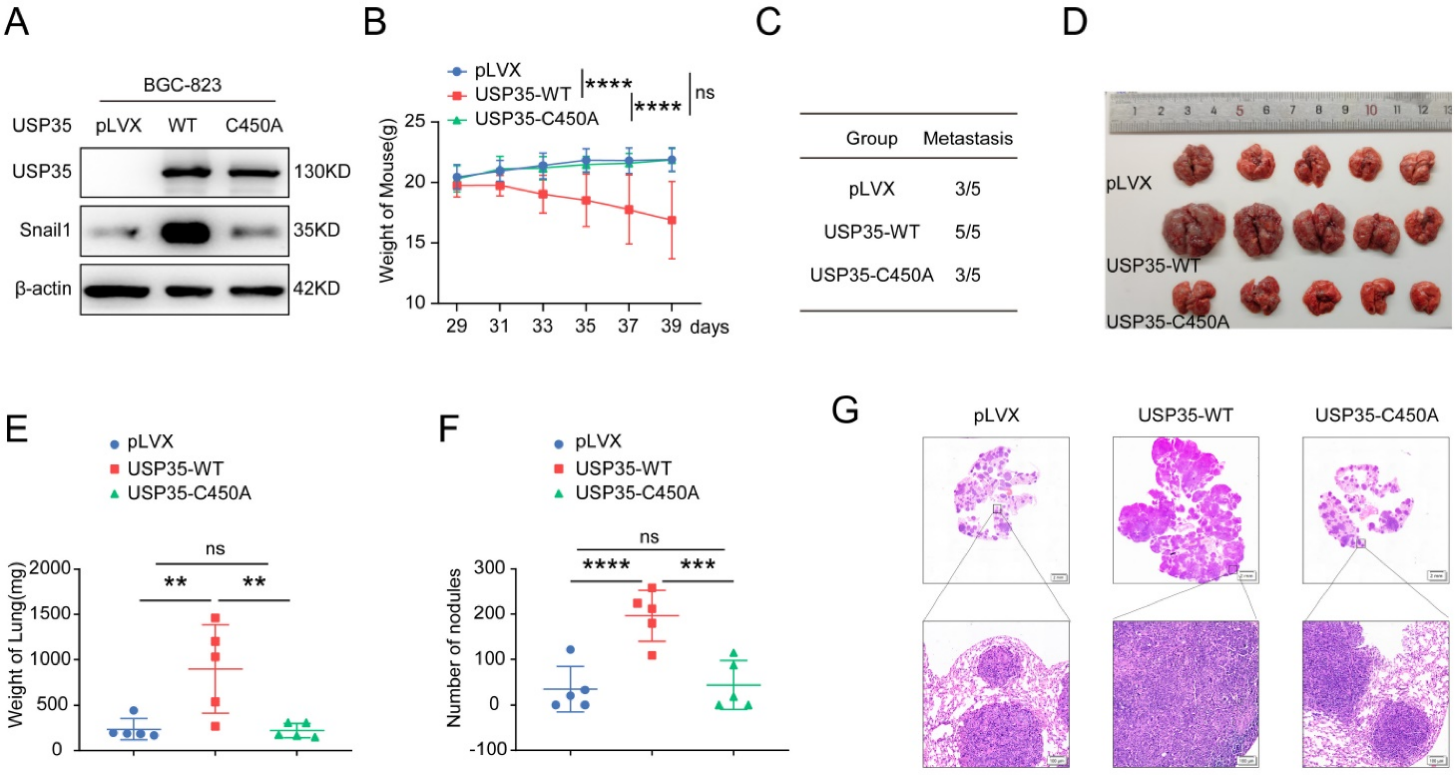
** USP35 promotes the metastasis of GC *in vivo.* A.** Western blot detection of Snail1 and USP35 protein level in BGC-823 cells stably overexpressing empty vector (pLVX), wild-type USP35 (WT) or USP35-C450A (C450A). **B.** Weight curve of the nude mice injected with BGC-823 cells stably overexpressing empty vector (pLVX), USP35-WT or USP35-C450A by tail vein. Data are expressed as the mean ± SD, two-way ANOVA test. **** *p* < 0.0001; ns: no significance. **C.** The number of nude mice with metastasis in the indicated groups. **D.** Images of nude mice lungs in the indicated groups. **E.** Quantification and statistical analysis of the lung weight of mice in (D). Data are expressed as the mean ± SD, unpaired Student *t-test*. ** *p* < 0.01; ns: no significance. **F.** Quantification and statistical analysis of the number of metastasis nodules on the surface of the nude mouse lung. Data are expressed as the mean ± SD, unpaired Student *t-test*. *** *p* < 0.001; **** *p* < 0.0001; ns: no significance. **G.** Representative images of H&E staining of the metastasis nodules in the lung tissues.

To further explore whether USP35 promotes cell metastasis *in vivo*, we collected BGC-823 cells stably overexpressing WT-USP35 or the USP35-C450A mutant (Fig. 7A) and performed experiments in nude mice by injecting the stably overexpressing cells into the tail vein. The mice were weighed every two days from day 29 to 39. The results demonstrated that the average weight of mice in the WT-USP35 group was much lower than that in the empty vector group. In contrast, there were no notable differences in the weight of nude mice between the control vector and USP35-C450A mutant groups (Fig. 7B). All five mice in the WT-USP35 group formed metastatic nodules on the surface of the lungs, while only three of the five mice in the control vector and USP35-C450A groups formed metastatic lung nodules (Fig. 7C). The lungs of mice in the WT-USP35 group were much larger and heavier than those in the empty vector group. In contrast, the USP35-C450A group showed no obvious differences from the empty vector group (Figs. 7D, E). Additionally, more metastatic nodules were observed in the WT-USP35 group than in the empty vector or USP35-C450A group (Figs. 7F, G). These results suggest that USP35 relies on DUB activity to promote GC metastasis *in vivo.*

### USP35 is positively correlated with Snail1 in clinical GC tissues

**Figure 8:**
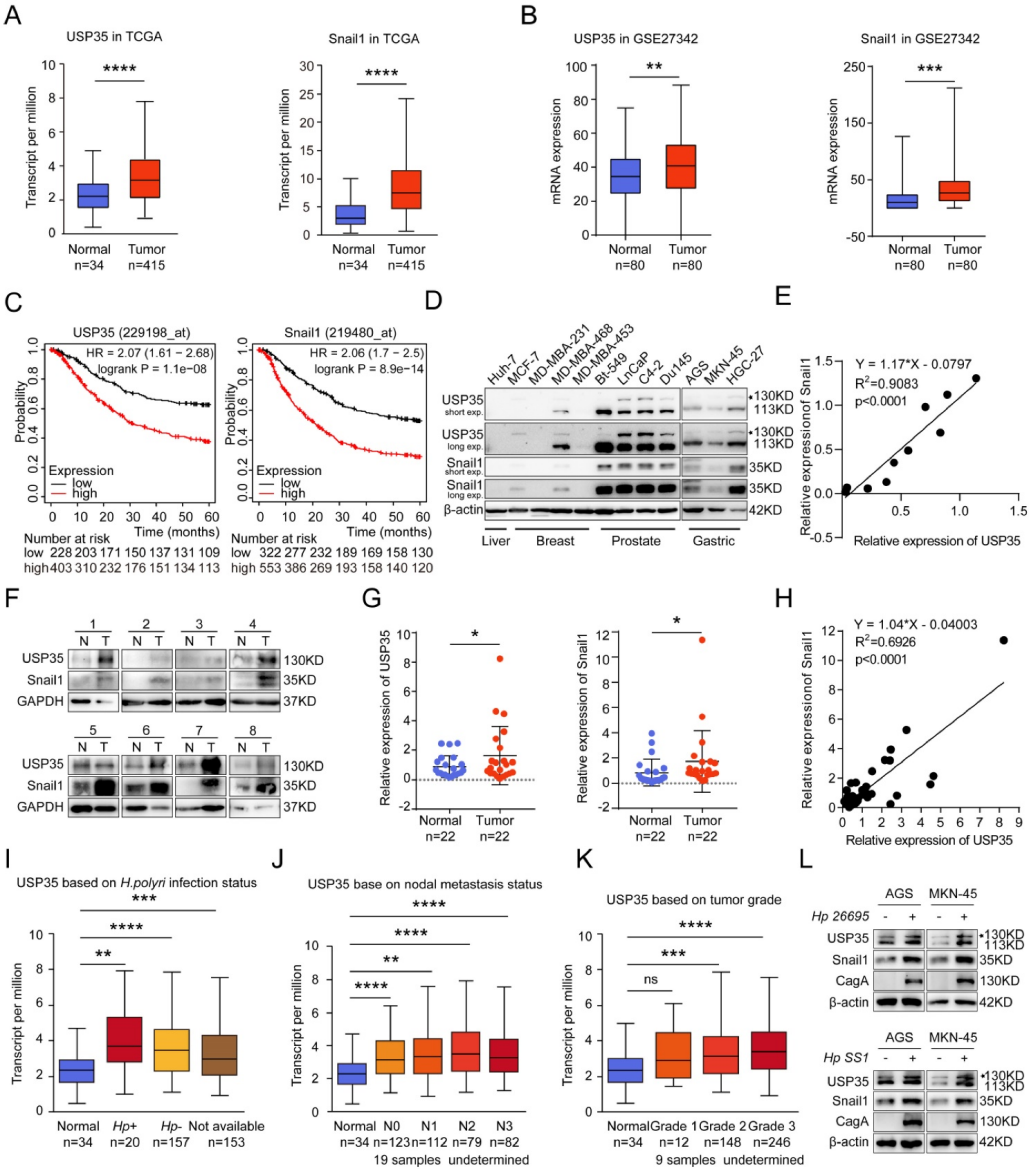
** USP35 and Snail1 are highly expressed in GC tissues and are positively correlated. A.** TCGA dataset analysis of mRNA expression levels of USP35 (left) and Snail1 (right) in GC and non-tumor tissues. **** *p* < 0.0001. **B.** GEO dataset (GSE27342) analysis of mRNA expression levels of USP35 and Snail1 in paired GC and adjacent non-tumor tissues. ** *p* < 0.01; *** *p* < 0.001. **C.** Kaplan-Meier Plotter database (http://kmplot.com/analysis/) analysis of the 5-year survival period of patients with GC in relation to the USP35 (left) and Snail1 (right) levels. **D.** Detection of USP35 and Snail1 protein expression in different tumor cell lines using Western blot. **E.** Quantitative analysis of USP35 and Snail1 protein expression in (D) using the ImageJ software. The correlation between USP35 and Snail1 in different tumor cells was analyzed. **F.** Western blot analysis of USP35 and Snail1 protein expression in 22 pairs of GC tissues and adjacent noncancerous tissues from patients (N: noncancerous tissues, T: tumor tissue). The representative results are presented. **G.** Quantitative and statistical analyses of USP35 (left) and Snail1 (right) protein expression in 22 pairs of GC and adjacent noncancerous tissues. * *p* < 0.05. **H.** Regression analysis of the correlation between USP35 and Snail1 protein expression in GC tissues (n = 22). **I, J, K.** Statistical analysis of USP35 expression in GC tissues based on* H. pylori* infection status (I), nodal metastasis stage (J), and tumor grade (K) according to the TCGA datasets. Data are the mean ± SD, unpaired Student's *t-test*. ** *p* < 0.01; *** *p* < 0.001; **** *p* < 0.0001, ns: no significance. **L.** Western blot analysis of USP35, Snail1, and CagA protein levels in GC cells infected with *H. pylori* 26695 or SS1.

To investigate whether the regulation of USP35 on Snail1 has clinical relevance, we analyzed the expression of Snail1 and USP35 using TCGA and GEO datasets and found that both USP35 and Snail1 were remarkably upregulated in GC tissues (Figs. 8A, B). The analysis of the Kaplan-Meier Plotter database demonstrated that higher expression of USP35 or Snail1 in GC tissues led to worse prognosis and lower 5-year survival period (Fig. 8C). To further examine the association between USP35 and Snail1 at the protein level, we measured the abundance of USP35 and Snail1 in various cancer cell lines, including liver, breast, prostate, and gastric cancers. The results indicated that USP35 expression was closely correlated with Snail1 protein abundance in these cell lines (Figs. 8D, E). We also investigated USP35 and Snail1 protein levels in clinical GC and adjacent noncancerous tissues. Our results showed that USP35 expression was elevated in 59% (13/22) of GC tissues and that Snail1 expression was high in 68.2% (15/22) of GC tissues. The representative results are shown in Fig. 8F. Statistical analysis indicated that both USP35 and Snail1 protein levels were significantly upregulated in GC tissues (Fig. 8G) and that USP35 was positively correlated with Snail1 in these GC samples (Fig. 8H). Further analysis of TCGA dataset showed that USP35 expression was associated with *H. pylori* infection, nodal metastasis status, and GC tumor grade (Figs. 8I-K). To further confirm the correlation between *H. pylori* infection and USP35 expression, we used *H. pylori* to infect GC cells and determined the protein levels of *H. pylori* virulence factor CagA and those of USP35 and Snail1. As shown in Fig. 8L, infection with *H. pylori* increased USP35 and Snail1 protein levels, indicating that *H. pylori* infection contributes to the upregulation of USP35 and Snail1.

## Discussion

As a reversible post-translational modification, ubiquitination is important in determining protein function and stability. This process can be reversed by a class of DUBs that remove the polyubiquitination chain from protein substrates. The dysregulation of DUBs may cause many diseases, particularly the occurrence and progression of tumors. DUBs can be subdivided into six families based on their sequence and domain conservation 36. The USP family is the largest DUB family. In this study, we initially investigated the expression levels of 51 USP family members in GC and noncancerous tissues using TCGA and GEO datasets. We discovered that 16 USPs were upregulated in GC tissues compared with their expression in noncancerous tissues in both databases (Supplementary Fig. S1). We further determined the correlation between these upregulated USPs and the prognosis of patients with GC using the Kaplan-Meier Plotter database and found that the higher expression of five USPs (USP5, USP13, USP10, USP21, and USP35) were closely associated with shorter OS and poorer prognosis in patients with GC (Supplementary Fig. S2).

Given that metastasis is the major reason for poor prognosis and cancer-associated deaths and that the EMT process is crucial in metastasis, we investigated whether the five USPs have regulatory effects on the expression of the major EMT transcription factor Snail1. Among the five USPs, USP35 has the most significant regulation on Snail1 expression in MKN-45 cells. USP35 overexpression increased the Snail1 protein levels, whereas USP35 depletion decreased Snail1 protein levels. Furthermore, the expression levels of USP35 and Snail1 were highly correlated in different tumor cells and clinical GC tissues. A couple of E3 ubiquitin ligases 13-20, 37 and DUBs 21-29, 37 have been identified to regulate the ubiquitination and degradation of Snail1. Among these DUBs, USP29 and USP13 have been reported to increase Snail1 protein levels in GC cells. However, there was no significant difference in the expression of USP29 between GC tissues and noncancerous tissues according to TCGA and GEO datasets (Supplementary Fig. S1). Therefore, we suppose that USP35 may be more suitable as a therapeutic target for GC than USP29. In addition, our results indicated that USP35 had a more obvious regulation on Snail1 in MKN-45 cells than USP13 (Fig. 1A).

In the present study, we observed that USP35 knockdown facilitated the degradation of Snail1, whereas USP35 overexpression delayed its degradation. The proteasome inhibitor MG132 blocked USP35 depletion-induced Snail1 degradation, suggesting that USP35 increases the stability of Snail1 by inhibiting its proteasomal degradation. Further studies verified that USP35 can interact with Snail1 and that the USP catalytic domain at the C-terminus of USP35 participates in its interaction with Snail1. Zhang et al. also revealed that the USP catalytic domain of USP35 is essential for its interaction with STING 34. These findings suggest that the USP catalytic domain of USP35 is important for mediating its interactions with target substrates. Ubiquitin contains seven internal lysine residues (K6, K11, K27, K29, K33, K48, and K63). In this study, we validated that USP35 can remove K6-, K11-, K27-, K33-, K48-, and K63- linked polyubiquitination chains from Snail1. The ubiquitination and degradation of Snail1 may be selectively regulated by different conditions such as DNA damage (acting on FBXL5) 15, hypoxia (acting on FBXL14) 17, and growth factors (acting on β-TrCP1) 14. However, the mechanism governing the DUB-mediated counteraction of specific E3 ligase-mediated ubiquitination and degradation of Snail1 remains unclear. Therefore, exploring how these different ubiquitin ligases and DUBs dynamically regulate Snail1 expression in cancer cells is advisable.

USP35 influences multiple biological functions, such as mitosis, ER stress-induced apoptosis, cisplatin-induced apoptosis, ferroptosis, mevalonate metabolism, cell proliferation, and the STING signaling pathway, by deubiquitinating various target substrates, including Aurora B 30, RRBP1 31, BIRC3 32, ferroportin 33, BRPF1 38, estrogen receptor α 39, and STING 34. However, the role of USP35 in EMT and tumor metastasis has not yet been reported. In the present study, we identified the regulatory role for USP35 in GC cell invasion and metastasis. We found that overexpression of wild-type USP35 promoted GC cell invasion and migration *in vitro* and facilitated tumorigenesis and metastasis *in vivo*. We also verified that USP35 promotes the proliferation and survival of GC cells, which is consistent with certain findings reported previously 30. These results demonstrate that USP35 serves as an oncogene in GC. Together with the data indicating that higher USP35 expression is associated with worse prognosis in patients with GC, our study suggests that USP35 may be exploited as a promising therapeutic target in GC.

The results based on the analysis of TCGA and GEO datasets showed that the expression level of USP35 is correlated with tumor grade, node metastasis, and *H. pylori* infection. Additionally, we confirmed that *H. pylori* infection upregulated the expression of USP35 and Snail1, indicating that *H. pylori* infection is an important factor leading to increased expression of USP35 and Snail1 in GC cells. Eliminating *H. pylori* may be an effective strategy to reduce the expression of USP35 and Snail1. However, the mechanism by which *H. pylori* infection causes the upregulation of USP35 remains to be explored.

**Figure 9:**
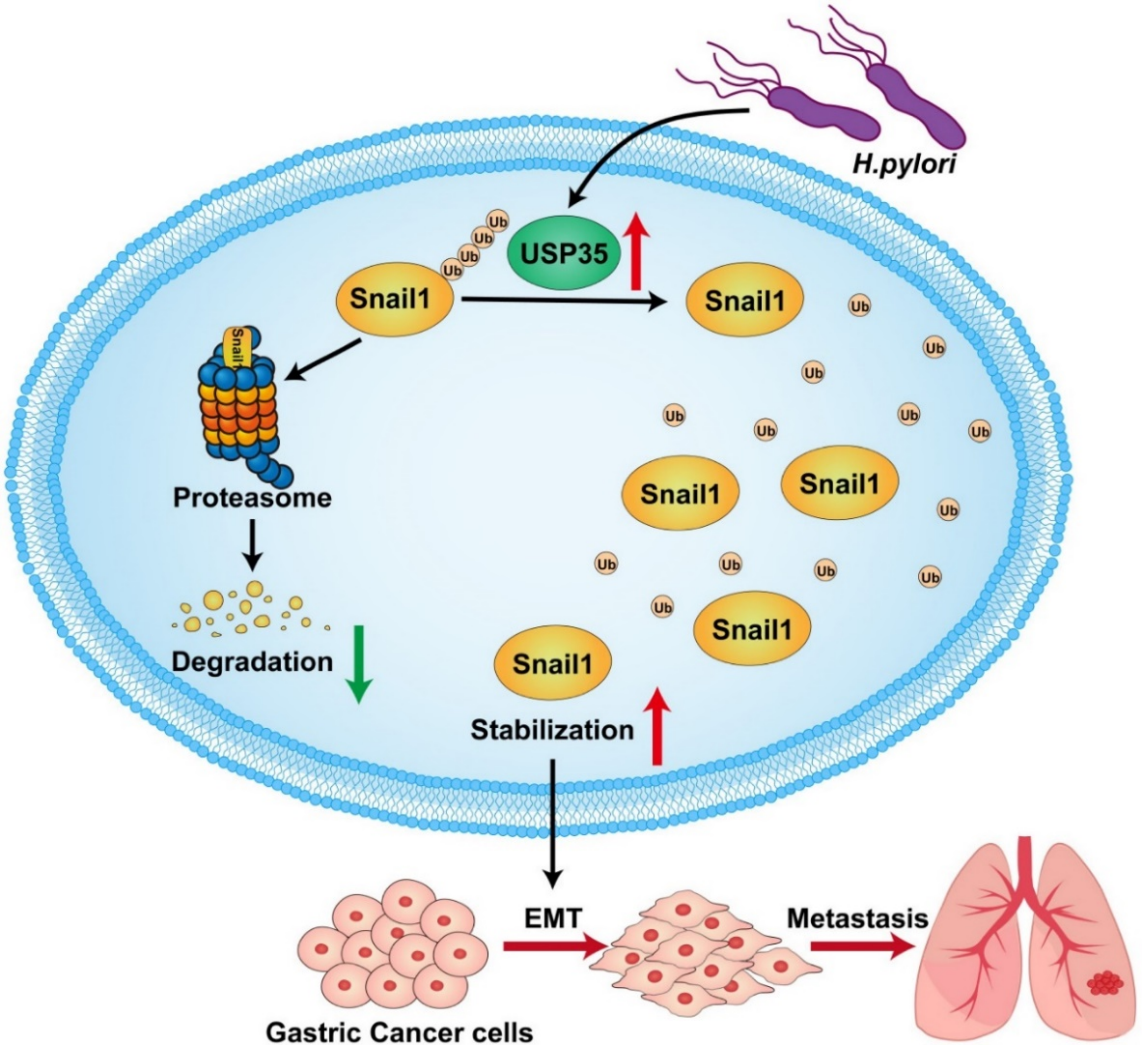
** Schematic diagram to summarize the findings in this study.** *H. pylori* -induced upregulation of USP35 deubiquitinates and stabilizes Snail1 to promote GC metastasis.

In summary, we found that USP35 is upregulated in GC tissues, which may partially result from *H. pylori* infection*.* High USP35 expression is associated with poor prognosis and short OS in patients with GC. USP35 interacts with and deubiquitinates Snail1, thereby increasing Snail1 protein levels. The USP domain of USP35 is necessary for interaction with Snail1. USP35 promotes GC invasion and migration, at least partially by regulating Snail1. Furthermore, USP35 expression closely correlates with Snail1 expression in GC tissues. The results are summarized in Fig. 9. These findings suggest that targeting USP35 may be a viable strategy for the effective treatment of GC.

## Supplementary Material

Supplementary materials and methods, figures and tables.

## Abbreviations

DUB: deubiquitinase
OS: overall survival
GC: gastric cancer
USP: ubiquitin specific proteases
TCGA: The Cancer Genome Atlas
GSEA: gene set enrichment analysis
EMT: Epithelial-mesenchymal transition
CHX: Cycloheximide
HE: hematoxylin-eosin
