# Supplementary material for: Ubiquitin-specific Protease 35 Promotes Gastric Cancer Metastasis by Increasing the Stability of Snail1
Source: Int J Biol Sci. 2024 Jan 12;20(3):953–67. doi: 10.7150/ijbs.87176 (PMC10797686; doi:10.7150/ijbs.87176)
Supplement: Supplementary file 1 — Supplementary materials and methods, figures and tables. [file ijbsv20p0953s1.pdf]

Supplementary information

Supplementary Materials and Methods

Cell culture and cell transfection

Human gastric carcinoma cell lines AGS, HGC-27, MKN-45, BGC-823 and embryonic kidney cell line HEK293T were routinely cultured in in HAM's F-12 or RPMI-1640 medium containing 10% fetal bovine serum (FBS, Biological Industries, USA) and 100 μg/mL of penicillin-streptomycin (Life Technologies, Carlsbad, CA, USA) at 37°C with 5% CO_2.

For cell transfection, we used Lipofectamine 2000 (11668019, Invitrogen, USA) to transfect siRNAs into GC cells and used Hieff Trans™ Liposomal Transfection Reagent (40802ES03, Yeasen, Shanghai, China) to transfect plasmids into HEK293T or GC cells. We performed the experiments in accordance with the manufacturer's instructions.

Clinical GC samples

Twenty-two pairs of clinical GC tissues and adjacent paracancerous tissues were obtained from patients with primary GC who underwent surgical resection at the Taian City Central Hospital (Taian, China). Detailed clinicopathological data are shown in Supplementary Table S4. All the patients provided written informed consent for the recommended procedures.

RNA extraction, reverse transcription, and qRT-PCR

Total RNA from GC cells was isolated using TriZol reagent. The cDNA was synthesized with random primers using the Evo M-MLV RT Mix Kit with gDNA Clean for qPCR (AG11728, Accurate Biotechnology Co., Ltd., Hunan, China). qRT-PCR was performed using SYBR green kit (Q311, ChamQ SYBR qPCR master Mix, Vazyme, Nanjing, China) and the Bio-Rad CFX96TM Real-Time PCR System. Primers for qRT-PCR were synthesized by Sangon Biotech (Shanghai, China). Primer sequences used in this study are listed in Supplementary Table S5. The relative mRNA expression levels were analyzed using the $2^{-\triangle\triangle}$Ct method. β-actin served as the internal control. All reactions were performed in triplicate.

EdU assay

The assay was performed following the instructions of the Cell-Light™ EdU Apollo®567 Kit (C10310-1, RiboBio, China). Briefly, the transfected GC cells were harvested and seed into 96-well plates (1×10^4 cells/ well). The next day, the cells were treated with 50uM EdU and incubated for 2 hours at 37 °C. Then, the cells were fixed with 4% paraformaldehyde (BL539A, Biosharp Life Science, Beijing, China) and stained with Hoechst 33342. The images were captured using a fluorescence microscope (Olympus, Tokyo, Japan).

CCK-8 assay

The transfected GC cells were added with 100 μL 10% CCK-8 solution and incubated for 2 hours at 37°C. The absorbance was measured at 450 nm with an Infinite M200

spectrophotometer (Tecan). The assay was performed in triplicate.

Scratch wound healing assay

GC cells transfected with siRNAs or expression vectors were cultured in 6-well plates. The scratch wound-healing assay was performed as described previously [35].

Transwell invasion and migration assays

The Transwell invasion and migration assays were performed as previously described [35]. Briefly, GC cells with different transfection were harvested, counted, and resuspended in serum-free RPMI-1640 medium. For the invasion assay, 6×10^4 GC cells were seeded into a Matrigel invasion chamber (3422; Corning, NY, USA), and for the migration assay, 4×10^4 cells were added to a Transwell chamber. The cells were incubated for 48 h and 36 h for the invasion and migration assays, respectively. After removing the cells that have not invaded or migrated on the upper surface of the polycarbonate membrane using cotton swabs, the membranes containing invasive or migratory cells on the lower side were fixed using 4% paraformaldehyde, stained with crystal violet (G1062, Solarbio Life Science, Beijing, China), and photographed under the microscope (CKX53，Olympus, Japan). The numbers of invading or migrating cells were counted in three independent experiments.

***H. pylori* cultures and infection assay**

The standard *H. pylori* strains *26695* and *SS1* were generous gifts from Dr. Jianzhong Zhang (Chinese Disease Control and Prevention Center, Beijing, China). The *H. pylori*

strains were cultured according to previously described method [35]. For *H. pylori* infection, GC cells (6 × 10^5) were seeded in 6-well plates and infected with *H. pylori* 26695 or SS1 at an MOI of 100:1 for 2 h. Cellular proteins were then extracted for Western blot.

Xenograft tumor experiment

Female BALB/c nude mice (4-5 weeks old) were purchased from Jiangsu Huachuang Sino Pharma Tech Co., Ltd. (Jiangsu, China). BGC-823 cells (5 × 10^5) stably overexpressing WT-USP35, USP35-C450A mutant, or control vector were injected into the tail veins of nude mice. Twenty-nine days later, the weight of the mice was monitored every two days. The mice were sacrificed on day 39. The lungs of nude mice were weighed and stained with hematoxylin and eosin (HE) stain. The mice experiments were approved by the Ethics Committee of the School of Basic Medical Sciences, Shandong University, and were performed in accordance with the guidelines of animal experiments at the Laboratory Animal Center of Shandong University.

Supplementary Figures

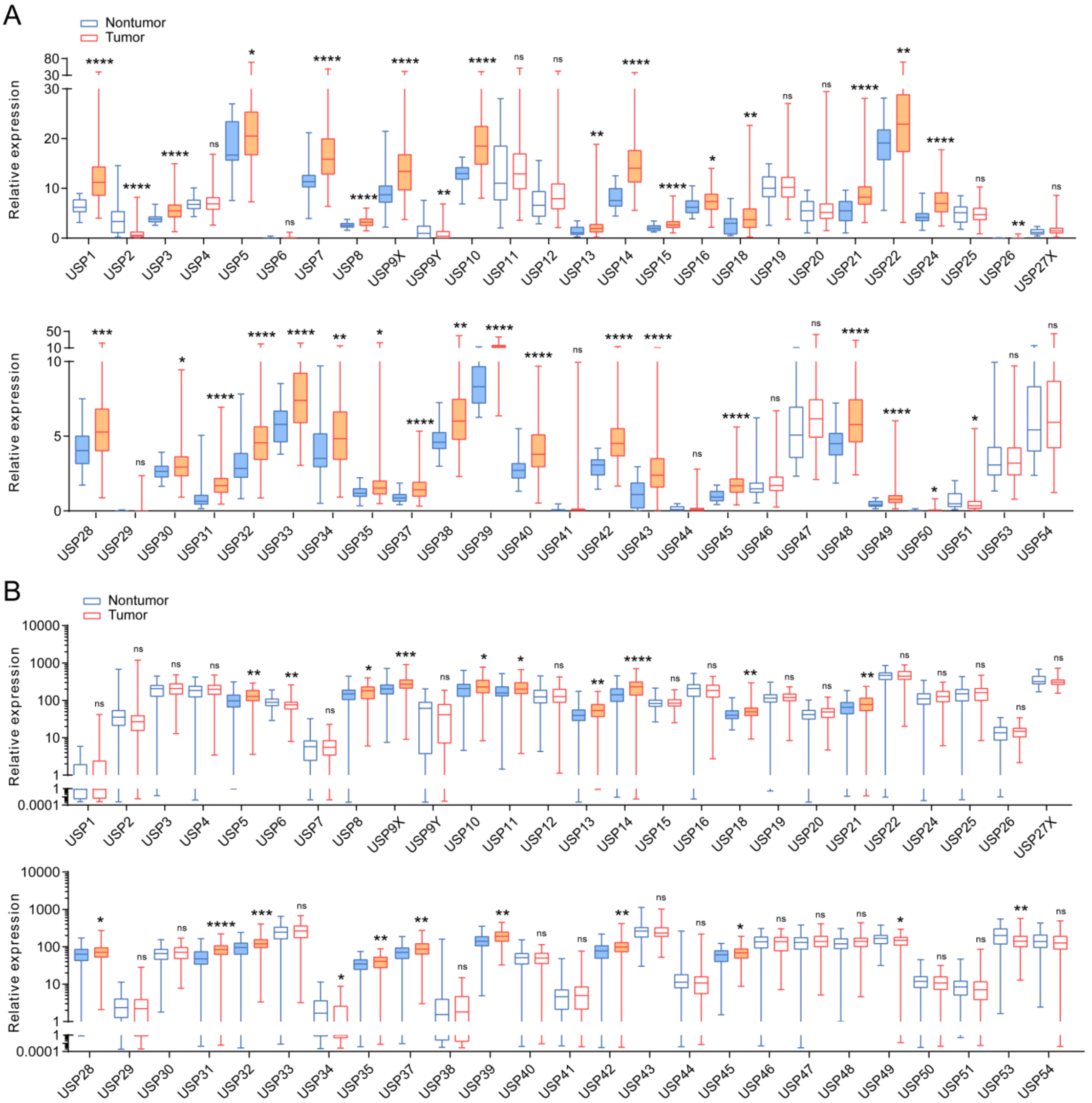

Figure S1. Analysis of mRNA expression levels of 51 USPs in GC tissues and noncancerous tissues based on the data from TCGA (A) and GEO (B) databases. Data were analyzed using Student's *t test*. * p < 0.05; ** p < 0.01; *** p < 0.001; **** p < 0.0001; ns: no significance.

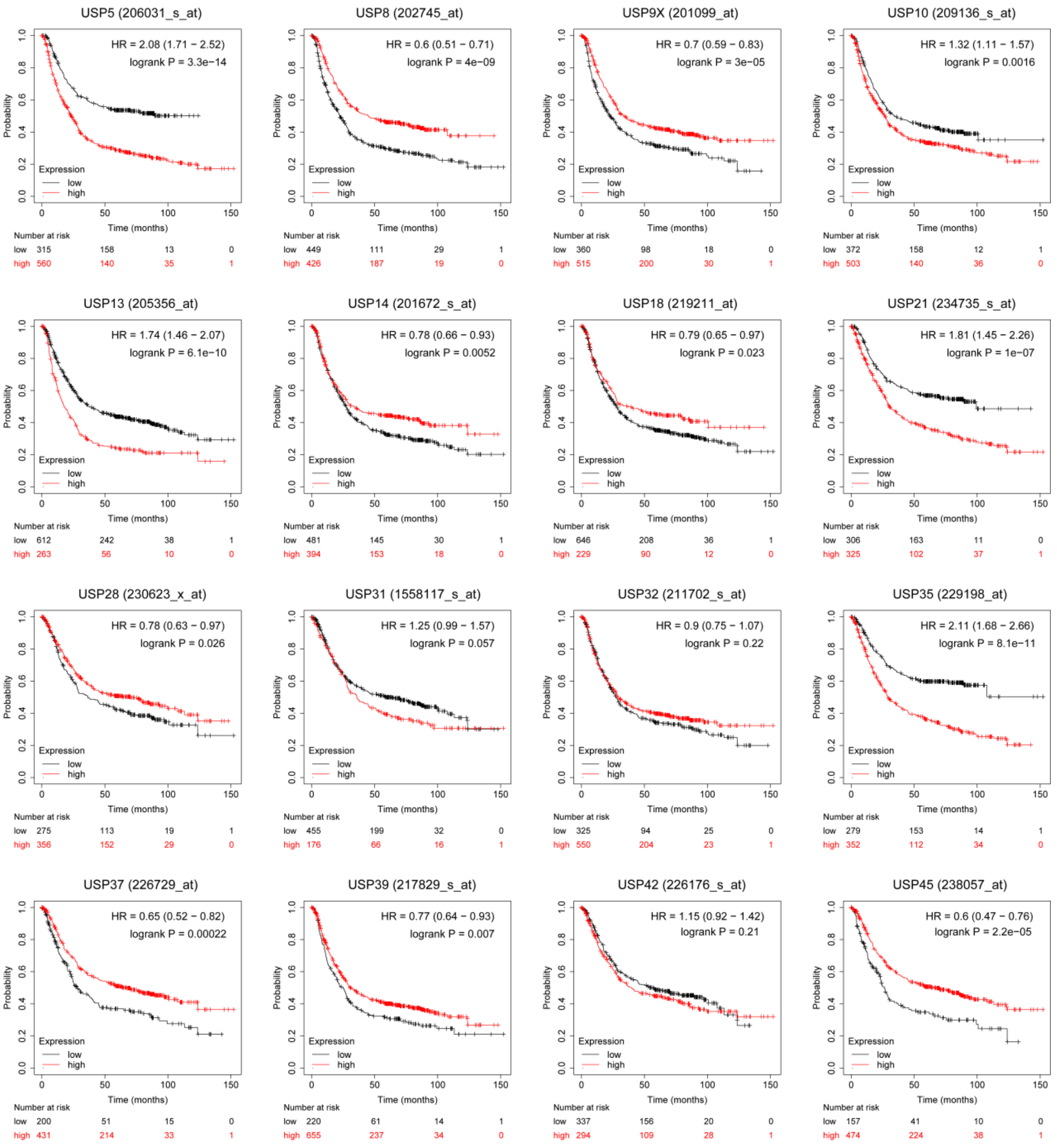

Figure S2. Kaplan–Meier Plotter database (http://kmplot.com/analysis/) was used to analyze the overall survival (OS) of patients with GC in relation to the 16 USPs.

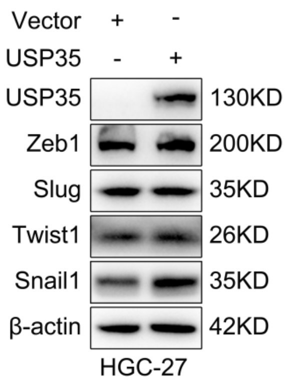

Figure S3. Western blot detection of EMT transcription factors in HGC-27 cells transfected with empty vector or USP35 expression vector.

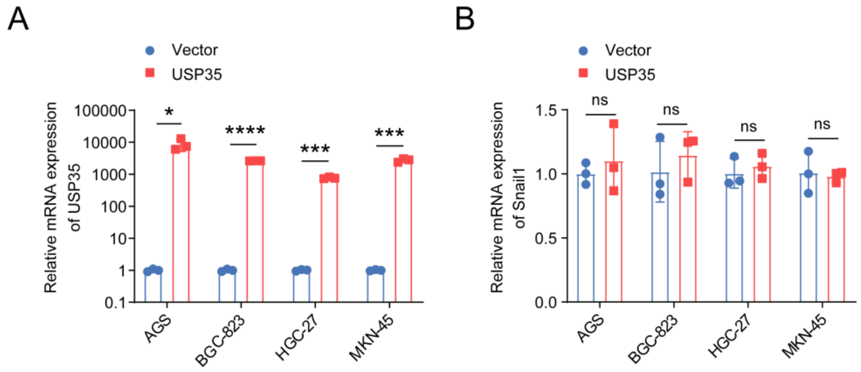

Figure S4. mRNA levels of *Usp35* and *Snail1* in GC cells transfected with empty vector or USP35 expression vector

A. qRT-PCR analysis of *Usp35* mRNA levels in different GC cells transfected with empty vector or USP35 overexpression vector.

B. qRT-PCR analysis of *Snail1* mRNA levels in different GC cells transfected with empty vector or USP35 overexpression vector. All data are represented as the mean ± SD from three independent experiments and analyzed with unpaired Student *t-test*. * $p < 0.05$; *** $p < 0.001$; **** $p < 0.0001$; ns: no significance.

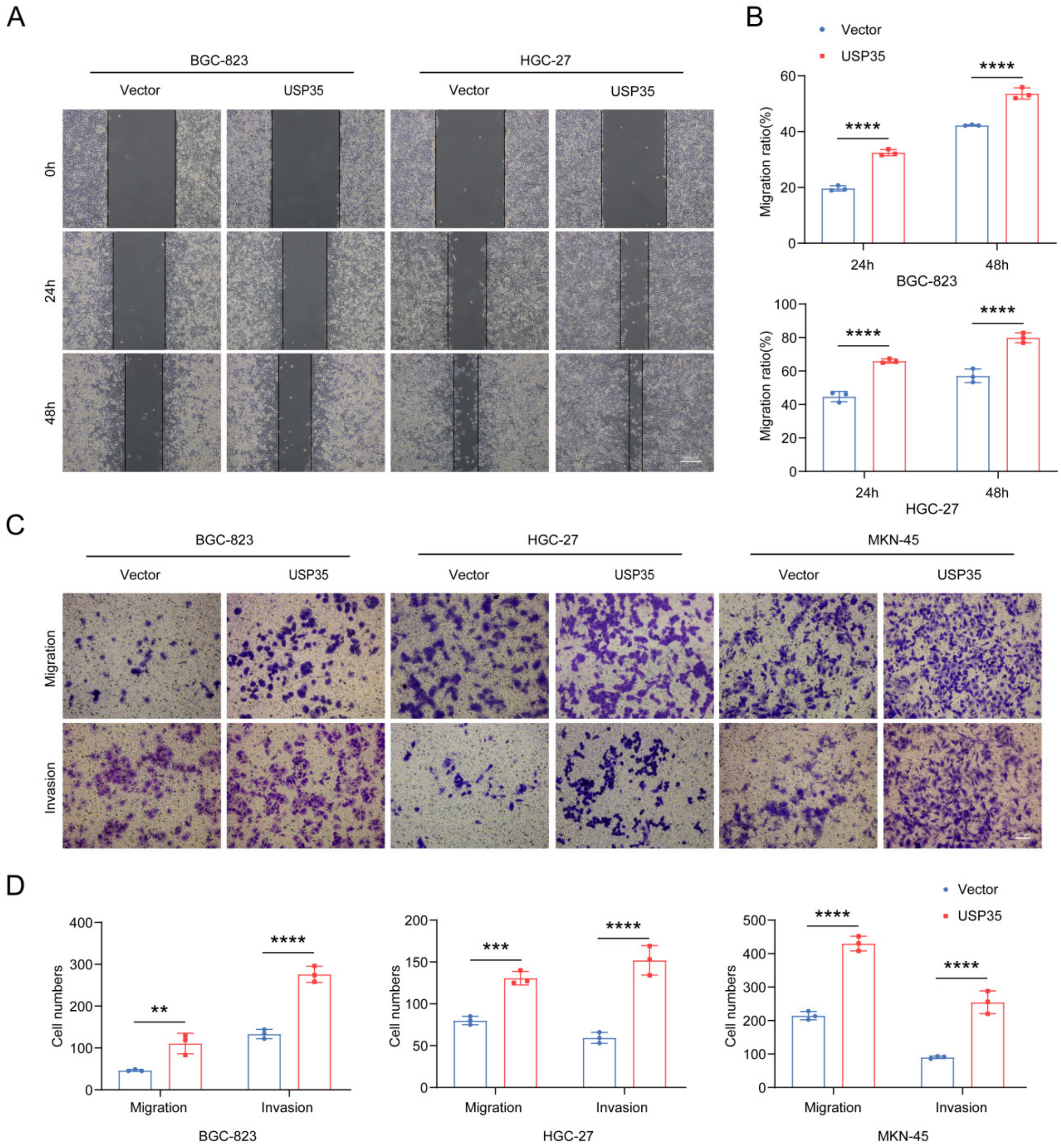

Figure S5. USP35 overexpression promotes GC cell invasion and migration

A. The scratch wound healing assay in GC cells transfected with USP35 expression vector or empty vector. Representative images were showed. Scale bar: 500 μm.

B. Statistical analysis of the cell migration rate in the scratch wound healing assay.

C. Transwell invasion and migration assays in GC cells transfected with USP35 expression vector or empty vector. The representative results were showed. Scale bar: 100 μm.

D. Statistical analysis of the cell numbers passing through the Transwell chamber in the transfected GC cells. All data are represented as the mean ± SD from three independent experiments and analyzed with unpaired Student *t-test*. ** $p < 0.01$; *** $p < 0.001$; **** $p < 0.0001$.

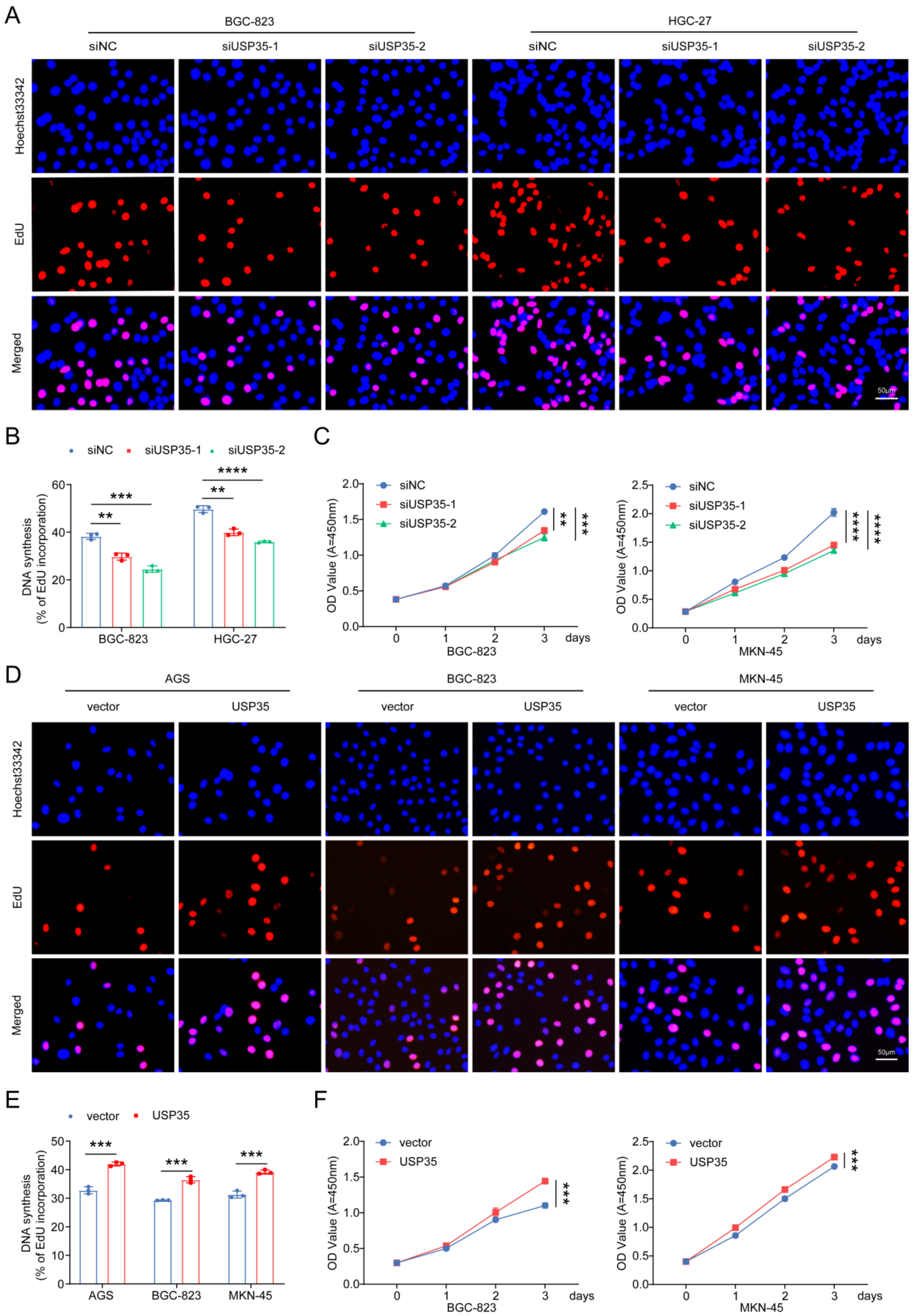

Fig. S 6

Figure S6. USP35 regulates the proliferation and survival of GC cells

A. Representative images of EdU assay in GC cells transfected with negative control

siRNA (siNC) or USP35 siRNA (siUSP35). Scale bar: 50 μm.

B. Statistical analysis of the percentage of EdU positive cells in GC cells with different transfection. The data are represented as the mean ± SD from three independent experiments and analyzed with unpaired Student *t-test.*

C. Statistical analysis of the results of CCK-8 assay in GC cells transfected with siNC or siUSP35. The data are represented as the mean ± SD from three independent experiments and analyzed with Two-Way ANOVA.

D. Representative images of EdU assay in GC cells transfected with empty vector (vector) or USP35 expression vector (USP35). Scale bar: 50 μm.

E. Statistical analysis of the percentage of EdU positive cells in GC cells with different transfection. The data are represented as the mean ± SD from three independent experiments and analyzed with unpaired Student *t-test.*

F. Statistical analysis of the results of CCK-8 assay in GC cells transfected with empty vector or USP35 expression vector. The data are represented as the mean ± SD from three independent experiments and analyzed with Two-Way ANOVA. ** $p < 0.01$; *** $p < 0.001$; ****$p < 0.0001$.

Supplementary Tables

Table S1. Primer sequences for vector construction in this study

Name	Primer sequence
USP35-C450A mutant	F:5'- GCAACACAGCCTATGTCAACAGCATCCTTCAGGC -3'
	R:5'- TGACATAGGCTGTGTTGCCCAGGTTGATGAGAC -3'
USP35 truncation mutant (1-753)	F:5'- CGGACATGTGGATCCCGGGTGGCATCCCTGTGA -3'
	R:5'- GGATCCACATGTCCGCTCCCCGGAGGGGATGGC -3'
USP35 truncation mutant (754-1018)	F:5'- GCGAATTCCGGCTCTGAGGGCTCCCGCTCCGTC -3'
	R:5'- AGAGCCGGAATTCGCGGCCGCAAGCTTAGCGTA -3'
USP35 truncation mutant (1-432)	F:5'- CCCCGGCTCGGATCCCGGGTGGCATCCCTGTGA -3'
	R:5'- GGATCCGAGCCGGGGATAGAAACCCGCCAGCTC -3''
USP35 truncation mutant (433-1018)	F:5'- GCGAATTCCATGGCCAAGTCAGACACGGGCAAG -3'
	R:5'- GGCCATGGAATTCGCGGCCGCAAGCTTAGCGTA -3'
USP35 truncation mutant (1-604)	F:5'- CCTCCTGAGGGATCCCGGGTGGCATCCCTGTGA -3'
	R:5'- GGATCCCTCAGGAGGAGGGAAGGCGAGAGAGAG -3'
Flag-tagged USP35 vector	F:5'-TACAAGGACGACGATGACAAGGGCGCCATGGACAAGA -3'
	R:5'-GTCATCGTCGTCCTTGTAGTCAAGCTTGTTCAGGTCC -3'
HA-tagged Snail1 vector	F:5'-TGGCCATGGAGGCCCGAATTCCCGCGCTCTTTCCTCGTC -3'
	R:5'-ACGCCGGACGGGTACCTCGAGTCAGCGGGGACATCCTGAG -3'

Table S2. siRNA sequences in this study

Name	siRNA sequence
USP35 siRNA-1	5'- AGAGCGAGCUGGCGGGUUU -3'
USP35 siRNA-2	5'- CAACAUCCUUUACCUACAG -3'
Negative control	5'- CCUAC AUCCCGAUCGAUGAUGUUGA-3'

Table S3. The information of antibodies in this study

Antibody	Source	Cat.No	Dilution ratio	
			Western blot	Co-IP
anti-USP35	Abcam	ab86791	1: 6000	1: 400
anti-Snail1	Cell Signaling Technology	3879	1: 1000	1: 400
anti-Zeb1	Cell Signaling Technology	3396	1: 1000	\
anti-Slug	Cell Signaling Technology	9585	1: 1000	\
anti-Vimentin	Cell Signaling Technology	5741T	1: 1000	\
anti-N-cadherin	Cell Signaling Technology	13116	1: 1000	\
anti-E-cadherin	Cell Signaling Technology	3195	1: 1000	\
anti-Twist1	Proteintech	25465-1-AP	1: 2000	\
anti-CagA	Santa Cruz Biotechnology	SC-17450	1: 500	\
anti-Flag	Sigma-Aldrich	F1804	1: 1000	1: 400
anti-Myc	Origene	TA150121	1: 1000	1: 400
anti-HA	Cell Signaling Technology	3724S	1: 1000	1: 400
Mouse IgG	Abmart	B30010	\	1: 400
Rabbit IgG	Abmart	B30011	\	1: 400
anti-GAPDH	ServiceBio	GB15004	1: 2000	\
anti-β-actin	Proteintech	66009-1-Ig	1: 7000	\

Table S4. Clinicopathologic characteristics of GC patients

No.	Gender	Age（yr）	TNM	Size（diameter, cm）
1	F	52	T4aN3bM1	4.5
2	F	59	T3N3bM1	5.5
3	M	55	T4aN3bM0	7.5
4	M	45	T4aN3aM1	10
5	M	73	T4aN3bM0	7
6	M	65	T3N0M0	7
7	M	62	T4N2M0	6
8	F	46	T4aN1M0	7
9	M	75	T3N3aM0	6
10	F	49	T4aN1M0	3
11	F	40	T3N3bM0	6
12	M	80	T4aN1M0	8
13	F	57	T3N3aM0	5.5
14	M	64	T3N2M1	5
15	M	66	T4N3M0	9
16	F	69	T3N3aM0	4
17	M	54	T4aN3aM0	7
18	F	65	T4aN3aM0	12
19	M	52	T4aN2M0	10.5
20	F	66	T3N1M0	8
21	M	59	T4N3M0	6.5
22	F	37	T4aN3aM1	13

Table S5. Primer sequences for qRT-PCR in this study

Name	Primer sequence
USP35 (126bp)	F:5’- TGCCATTAGCAGGATGATTGA -3’
	R:5’- AGCGAAACCTCGATCAAGATG -3’
Snail1 (211bp)	F:5’- GGAAGCCTAACTACAGCGAGCT -3’
	R:5’- TCCCAGATGAGCATTGGCA -3’
β-actin (177bp)	F:5’- GAAGTGTGACGTGGA CATCC -3’
	R:5’- CCGATCCACACGGAGTACTT -3’
